# Maternal smoking during pregnancy and offspring psychiatric disorder: a longitudinal birth cohort study

**DOI:** 10.1007/s00127-021-02094-w

**Published:** 2021-05-04

**Authors:** Ross Brannigan, Antti Tanskanen, Matti O. Huttunen, Mary Cannon, Finbarr P. Leacy, Mary C. Clarke

**Affiliations:** 1 Psychology Department, Royal College of Surgeons in Ireland, Room 2, -1 Beaux Lane House, Lower Mercer Street Dublin 2, Dublin, Ireland; 2 Department of Psychiatry, Royal College of Surgeons in Ireland, Dublin, Ireland; 3 Department of Clinical Neuroscience, Karolinska Institute, Stockholm, Sweden; 4 Department of Mental Health, National Institute for Health and Welfare, Helsinki, Finland; 5 Data Science Centre, Royal College of Surgeons in Ireland, Dublin, Ireland

**Keywords:** Prenatal Stress, Prenatal Smoking, Psychiatric Disorders, Epidemiology

## Abstract

**Background:**

There is evidence that prenatal stress and smoking during pregnancy both independently increase the risk of offspring psychopathology. Here we examine whether increased levels of self-reported stress is associated with increased smoking in a population of pregnant women, and whether prenatal smoking is associated with offspring psychiatric diagnoses independent of prenatal stress exposure.

**Method:**

Using a longitudinal birth cohort, we used ordered logistic regressions to examine associations between maternal stress and smoking during pregnancy. We then used logistic regression analyses to examine associations between prenatal smoking and later offspring psychiatric disorders.

**Results:**

A dose–response relationship was found between maternally reported stress and smoking during pregnancy. Pregnant women reporting severe stress were more likely to smoke compared to both the moderate stress and no stress groups, and those reporting moderate stress were significantly more likely to smoke compared to the no stress group. Smoking more than 5 cigarettes daily during pregnancy increased the risk of offspring personality disorder (OR 3.08, 95% CI 1.60–5.94) as well as developing any Axis 1 psychiatric disorder, inclusive of mood, anxiety and psychotic disorders (OR 1.45, 95% CI 1.04–2.04). After adjusting for parental psychiatric history and maternal self-reported stress during pregnancy, associations between smoking more than 5 cigarettes daily when pregnancy and offspring personality (OR 2.58 95% CI 1.32–5.06) disorder remained.

**Conclusion:**

Exposure to cigarette smoking during gestation could impact a child’s mental health. Smoking during pregnancy is a prime target for preventative interventions as unlike most other environmental risk factors, it is very amenable to change.

## Introduction

Maternal smoking during pregnancy has been previously associated with various externalizing disorders including conduct disorder [1, 2], and oppositional defiant disorder [2]. Additionally, Ashford et al. [3] showed in a sample of 397 individuals that those exposed to smoking during pregnancy had an increased risk of both internalizing and externalizing symptoms at ages 5, 10, 11 and 18. However, there is a paucity of research examining the association between maternal smoking during pregnancy and diagnosed adult psychiatric disorder in the offspring, especially internalizing disorders. Here, we aim to fill this gap in the literature by investigating associations between prenatal smoke exposure and offspring diagnosed psychosis, mood disorder, anxiety disorder or personality disorder up to age 30 in a longitudinal cohort study with prospectively collected data.

Maternal stress during pregnancy has also been associated with increased risk of various psychiatric diagnoses in the exposed offspring [4, 5]. Previously we have shown that prenatal stress increases the odds of offspring psychiatric diagnoses including mood and personality disorder [4, 5]. Stress and smoking have been previously associated whereby exposure to stress increased the desire to smoke in a mixed gender sample of smokers [6]. This association has also been reported regarding work-related stress, which has been shown to be associated with increased smoking [7]. Therefore, it is possible that the association between prenatal smoking and offspring psychiatric diagnoses is driven by exposure to prenatal stress.

Additionally, it is worth pointing out that associations between prenatal smoking and psychiatric disorders could also be product of residual confounding from a variety of areas, including socioeconomic, genetic and environmental [8]. One example of this related specifically to schizophrenia and bipolar disorders have been shown to be a product of genetic confounding rather than a causal association [9], while we do not have genetic information, we have attempted to account for potential genetic confounding by controlling for maternal and paternal psychiatric history.

In this study, we first examined whether women who reported stress during pregnancy were more likely to smoke. Second, we examined whether there were associations between prenatal smoking and later offspring psychiatric disorder, while controlling for prenatal stress and maternal and paternal psychiatric history in a longitudinal birth cohort.

## Methods

### Participants

This study used a subsample (*N* = 3633) of the Helsinki Temperament Birth Cohort (*N* = 6468), a birth cohort inclusive all of births in the greater Helsinki areas between June 1975 and 1976. This subsample included all expectant women who returned at least one well-being questionnaire during pregnancy. There were no differences in psychiatric outcomes between those included and not included in the subsample (*t* (6466) = 1.08, *P* = 0.28).

### Data collection

#### Prenatal stress

Self-reported maternal mental stress was collected every month during pregnancy as part of the well-being assessment carried out during antenatal clinic appointments. Participants completed a questionnaire which asked whether they were mentally stressed over the past month with three responses possible: none, moderately and severely. A modal measure of prenatal stress was created by identifying the most frequently reported stress level across each of individual’s questionnaires. Due to variation in the number of questionnaires completed, a modal measure is considered to be more representative of experienced stress than the mean as it captures the pregnant woman’s state of stress for the largest period of time during pregnancy.

#### Prenatal smoking

Information regarding prenatal smoking was collected using the same well-being assessments collected monthly during their antenatal clinic appointments by the clinic nurses. This questionnaire asked women if they smoked and how many cigarettes per day if so. The amount of smoking during pregnancy item had 3 possible responses: none, less than 5 daily and more than 5 daily.

A modal model of smoking was also used, as due to the variation in the number of questionnaires completed, the modal model is considered to best represent smoking behavior during pregnancy.

#### Outcome—psychiatric disorders

Outcomes for this study were ascertained through the linkage of the birth cohort data to the Finnish Hospital Discharge Register (FHDR) and the Finnish Population Register (FPR). Outcomes examined in this study were collected when the offspring were age 30 and they included any in-patient lifetime diagnosis of a psychotic, depressive, anxiety or personality disorder, as well as an any Axis 1 disorder category inclusive of all psychotic, depressive and anxiety disorders.

#### Confounders

We adjusted for parental psychiatric history as a potential confounding variable. Parental psychiatric history included both lifetime paternal and maternal psychiatric diagnoses ascertained through the FDHR.

Second, we adjusted for the total number of well-being questionnaires collected per woman as there was some variation in the number of antenatal clinic visits across women.

#### Missing data

Of the 3633 individuals in the original sample with pregnancy well-being information, 14 individuals did not have stress information, leaving 3619 individuals in the final sample used for the examination of the association between prenatal stress and smoking during pregnancy.

Additionally, there were 73 cases of missing data regarding paternal psychiatric history. These individuals were omitted from multivariate models, leaving 3546 individuals in the final adjusted sample used to examine associations between smoking during pregnancy and offspring psychiatric diagnoses.

### Data analysis

Stata 15.1 [10] was used to perform logistic and ordered logistic regression analyses.

Ordered logistic regressions were run to examine associations between maternal prenatal stress and maternal smoking during pregnancy. Logistic regressions were used to examine associations between maternal smoking during pregnancy and offspring psychiatric disorders up to age 30.

## Ethical approval

Ethical approval for the collection and use of the Helsinki Temperament Cohort, and the corresponding register information was granted by the Ministry of Social Welfare and Health (Approval number = 1430/69/82).

## Results

Of the 3619 individuals with complete data on prenatal stress and maternal smoking during pregnancy, 2570 (71%) individuals reported not smoking at all, 408 (11%) reported smoking less than 5 cigarettes daily and 641(18%) reported smoking more than 5 cigarettes daily. Regarding prenatal stress, 1647 (46%) women reported no mental stress throughout pregnancy, 1853 (51%) individuals reported moderate mental stress and 119 (3%) reported severe mental stress. The mean age of expectant mothers at the time of birth of the index child was 27.13, with an SD of 4.35. Of the expectant mothers, 223 had a previous or present psychiatric disorder, with 207 cases in the expected fathers. The median number of prenatal questionnaires returned was six with the first and third quartiles being 4 and 8.

Any maternal stress during pregnancy was associated with maternal smoking during pregnancy (OR 1.66, 95% CI 1.46–1.88). When looking at levels of stress, we see a dose–response relationship whereby the greater the level of stress experienced the greater the odds of smoking. Women who experienced moderate mental stress had 1.5 times the odds of smoking compared to those who reported no stress (OR 1.57, 95% CI 1.35–1.82), and women who reported severe mental stress during pregnancy had greater than 3-times the odds of maternal smoking compared to the no stress group (OR 3.23, 95% CI 2.28–4.56) and 2 times the odds compared to the moderate stress group (OR 2.05, 95% CI 1.46–2.89).

There were 204 cases of Axis 1 psychiatric disorders, with 55 cases of psychotic disorder, 69 cases of mood disorder and 122 cases of anxiety disorder in the offspring. There were also 40 cases of personality disorder. Unadjusted logistic regression models examining associations between prenatal smoking and offspring psychiatric disorders in adulthood can be seen in Table 1, using the no smoking group as the reference group.

**Table 1:** The association between prenatal smoking and later offspring psychiatric disorders

Diagnosis	Any smoking (OR(95% CI))	Less than 5 cigarettes daily (OR(95% CI))	More than 5 cigarettes daily (OR(95% CI))
Any Axis 1 disorder	1.20 (0.89–1.63)	0.82 (0.50–1.36)	1.46 (1.04–2.04)*
Psychotic disorder	1.19 (0.67–2.10)	0.85 (0.33–2.17)	1.40 (0.74–2.65)
Depressive disorder	1.31 (0.79–2.16)	0.84 (0.35–1.98)	1.61 (0.92–2.80)
Anxiety disorder	1.24 (0.85–1.82)	1.01 (0.56–1.83)	1.39 (0.90–2.16)
Personality disorder	2.23 (1.19–4.16)*	0.90 (0.27–3.03)	3.08 (1.60–5.94)*

^*^P < 0.05

After adjustment for maternal mental stress during pregnancy, parental psychiatric history, and total number of prenatal questionnaires returned, smoking more than 5 cigarettes daily remained significantly associated with later offspring personality disorder (OR 2.37, 95% CI 1.19–4.73)). The association between smoking more than 5 cigarettes daily during pregnancy and any Axis 1 disorder in the offspring became weaker (Ajd.OR 1.32, 95% CI 0.93–1.87); however, a strong trend still exists, suggesting that there are links between exposure to prenatal smoking and later psychiatric disorder in the offspring. Adjusted odds rations can be seen below (Table 2).

**Table 2:** Adjusted odd rations for associations between prenatal smoking and later offspring psychiatric disorders (post adjustment for maternal reported prenatal stress, maternal and paternal psychiatric diagnosis and total prenatal questionnaires returned)

Diagnosis	Any smoking (OR (95% CI))	Less than 5 cigarettes daily (OR (95% CI))	More than 5 cigarettes daily (OR (95% CI))
Any Axis 1 disorder	1.07 (0.78–1.46)	0.68 (0.40–1.16)	1.32 (0.93–1.87)
Psychotic disorder	1.04 (0.57–1.87)	0.79 (0.31–2.04)	1.19 (0.61–2.33)
Depressive disorder	1.04 (.61–1.77)	0.61 (0.24–1.55)	1.32 (0.74–2.36)
Anxiety disorder	1.18 (0.80–1.76)	0.92 (0.49–1.71)	1.35 (0.86–2.12)
Personality disorder	1.77 (0.92–3.40)	0.80 (0.23–2.70)	2.37 (1.19–4.73)*

*P* < 0.05

## Discussion

Our initial analysis showed that women who experience mental stress during pregnancy were more likely to smoke during pregnancy, with a dose–response relationship being observed, higher levels of mental stress during pregnancy led to higher odds of smoking. Studies examining stress and smoking in the general adult population have shown that desire to smoke is increased during times of increased stress [6], as well as associations between increased smoking and work-related stress [7]; however, this is the first study showing these associations in a population of pregnant women.

Second, our results show that smoking during pregnancy is associated with an increased risk of personality disorder in offspring, mainly driven by those smoking 5 or more cigarettes daily. We have shown that this association is independent of maternal stress during pregnancy, which is an important confounder, as stress increases both the risk of smoking, and of offspring poor mental health [4, 5]. Additionally, our results show strong trends between smoking 5 and more cigarettes during pregnancy and offspring development of any Axis 1 psychiatric disorder. Although this association became weaker after accounting for prenatal stress, the strong trend indicates that this association is worthy of further investigation.

These results add to the literature on the association between maternal smoking during pregnancy and externalizing disorders [1, 2], as well as symptoms of both internalizing and externalizing disorders [3], in the exposed offspring. Early intervention in personality disorder has been show to improve long terms outcomes [11]. Increased public health efforts to support women during pregnancy including with smoking cessation may reduce risks not just for physical adverse outcomes but also potentially for mental health outcomes.

### Potential mechanisms

The mechanisms underpinning the association between gestational smoke exposure and later psychiatric disorders and personality disorders are unclear. Knopik et al. [12] suggested an impact of smoking on fetal development and reported epigenetic effects of maternal smoking during pregnancy, such as altered DNA methylation and dysregulated expression of microRNA.

Second, cigarette smoke exposure during gestation is associated with fetal growth restriction. It has been shown that cigarette smoking is a risk factor for low birth weight; with one study reporting it is solely responsible for 7% of the risk [13] and another study showing that prenatal smoking was associated with increased odds of offspring being born with shorter body lengths for gestational age [14]. Low-birth weight has previously also been associated with increased risk for various psychiatric disorders, including depression and schizophrenia. It is possible that the effects of prenatal smoking are mediated through an impact on birth weight.

Finally, it is also possible that these associations are a product of other factors such as genetic or socioeconomic factors. Socioeconomic factors such as maternal education, family factors and social support. Maughan et al. [15] showed that mothers who smoked during pregnancy were more likely to be from socially disadvantaged households, with associations between prenatal smoking and offspring conduct problems being explained by social disadvantage and maternal depression. Additionally, Quinn et al., [9] showed in a large Swedish sample, that significant associations between prenatal smoking and offspring schizophrenia and bipolar disorders weaken when adjusting for familial confounding and dissipate when using a sibling comparative approach, supporting the hypothesis that these associations are driven by genetic confounding rather than being a causal association.

### Strengths and limitations

A major strength of this study is the register outcome data, which has been shown to have high diagnostic reliability [16]. A second strength is the prospective design by which the smoking data were collected, which ensured the results are not subject to recall bias.

The main limitation of this study is the possibility of unmeasured confounding, as we do not have data on socioeconomic factors such as maternal education and household income, which would likely impact both mental stress levels and smoking behavior during pregnancy. However, these factors are less relevant in this population as the exposure information was collected in 1975, before there was a population-wide understanding of the health dangers of smoking and before health warnings regarding smoking were widely circulated. It is likely that the confounding effects of socioeconomic factors are not as pertinent to this study as they would be to a similar study carried out in more recent years. This assumptions can be observed in general smoking rates, with Jemal et al., [17] reporting reductions in smoking across 30 states in the US, with only one state showing an increased smoking rate (approx. 1% per year) between 1975 and 2005. More recently, Johnstone et al., [18] showed smoking prevalence among various adolescent groups at its lowest in 2018 since 1974. While these are general populations, not-specific to pregnancy, they highlight the decrease in smoking prevalence in recent times.

An additional limitation lies with a lack of potential genetic confounders. Our adjustment for partental psychiatric history is not optimal, rather future studies should aim to control for genetic confounding through the use of family-based approaches or within family comparisons (e.g. sibling comparisons) as they provide associations which are usually independent from genetic, environmental or upbringing factors [9, 19].

## Conclusion

Smoking during pregnancy was associated with an increased risk of personality disorder in offspring, independent of the effects of prenatal stress and parental psychiatric history. There was also an association between smoking during pregnancy and any Axis 1 psychiatric disorder, which warrants further investigation. These results suggest that public health emphasis should be focused on the awareness and prevention of smoking during pregnancy for mental as well as physical health in the exposed offspring.
